# Soil-derived cellulose-degrading bacteria: screening, identification, the optimization of fermentation conditions, and their whole genome sequencing

**DOI:** 10.3389/fmicb.2024.1409697

**Published:** 2024-07-10

**Authors:** Degao Ma, Haoyu Chen, Duxuan Liu, Chenwei Feng, Yanhong Hua, Tianxiao Gu, Xiao Guo, Yuchen Zhou, Houjun Wang, Guifeng Tong, Hua Li, Kun Zhang

**Affiliations:** ^1^Yangzhou Environmental Monitoring Center of Jiangsu Province, Yangzhou, China; ^2^College of Plant Protection, Yangzhou University, Yangzhou, China; ^3^Department of Pharmacy, Medical School of Yangzhou University, Yangzhou University, Yangzhou, China; ^4^College of Engineering, Nanjing Agricultural University, Nanjing, China; ^5^Joint International Research Laboratory of Agriculture and Agri-Product Safety of Ministry of Education of China, Yangzhou University, Yangzhou, China

**Keywords:** cellulose degrading, soil bacterial, Cellulase activity assays, whole genome sequencing, glycoside hydrolase family genes

## Abstract

Straw cellulose is an abundant renewable resource in nature. In recent years, the conversion of cellulose from waste straw into biofuel by specific microorganisms’ fragmentation has attracted extensive attention. Although many bacteria with the ability to degrade cellulose have been identified, comprehensive bioinformatics analyses of these bacteria remain limited, and research exploring optimal fragmentation conditions is scarce. Our study involved the isolation and screening of bacteria from various locations in Yangzhou using carboxymethyl cellulose (CMC) media. Then, the cellulose-degrading bacteria were identified using 16S rRNA and seven candidate bacterial strains with cellulose degrading ability were identified in Yangzhou city for the first time. The cellulase activity was determined by the 3,5-dinitrosalicylic acid (DNS) method in different fragmentation conditions, and finally two bacteria strains with the strongest cellulose degradation ability were selected for whole genome sequencing analysis. Sequencing results revealed that the genome sizes of *Rhodococcus wratislaviensis* YZ02 and *Pseudomonas Xanthosomatis* YZ03 were 8.51 Mb and 6.66 Mb, containing 8,466 and 5,745 genes, respectively. A large number of cellulose degradation-related genes were identified and annotated using KEGG, GO and COG analyses. In addition, genomic CAZyme analysis indicated that both *R. wratislaviensis* YZ02 and *P. Xanthosomatis* YZ03 harbor a series of glycoside hydrolase family (GH) genes and other genes related to cellulose degradation. Our finding provides new options for the development of cellulose-degrading bacteria and a theoretical basis for improving the cellulose utilization of straw.

## Introduction

Straw, a renewable resource rich in organic matter and possessing high recycling value, plays a crucial role in sustainable agricultural practices (Li, 2010). Integrating straw back into the fields has proven to be an effective environmental strategy, particularly for farmers in the Yangtze River basin. This approach enhances the soil’s physical and chemical properties, increases organic matter content, and ultimately boosts crop yields (Hui-juan and Xia-li, 2018). However, soils enriched with organic matter can exacerbate airborne vegetable diseases, indirectly diminishing the quality of agricultural products through toxic metabolites (Xie et al., 2022). As the grain production increases yearly in China (Wang et al., 2018), so does the straw production. Large amount of the straw was left after harvesting of the crops in the field, and only a small portion of crop straw was further processed for animal feed, household fuel, soil amendment, industrial material (Shi et al., 2023). Most crop straw was wasted in the field, which named for returning the straw back to the field for degradation and production for organic matter. Straw does not decompose easily under natural conditions for their dense cross-linking of chemical bond between the cellulose, hemicellulose, and lignin (Yang et al., 2022). Direct burning of straw is the simplest and most common method, but the smoke and dust produced by burning seriously increase greenhouse gas emissions (Han et al., 2023), and damage the atmospheric environment where people lives (Xin-Yu et al., 2020). Consequently, the development of efficient straw degradation strategies becomes imperative for both high-quality agricultural advancement and environmental preservation (Liu and Han, 2021).

Straw is composed of lignin, cellulose and hemicellulose, making it resistant to degradation under natural conditions (Jin and Chen, 2007). Cellulose is a chain-like organic polymers that linking glucose unit by β-1,4 glycosidic linkages, and is the most abundant polysaccharide on the Earth (McDonald et al., 2012). Cellulose molecules exist in the form of molecular beams, aggregated in the order of microfibrils, forming a fine crystal structure, which is difficult to degrade by chemical reagents (Chen, 2011). However, the microbial decomposition of cellulose offers benefits such as cost-effectiveness, minimal harmful by-products, and relatively simple processing conditions (Asgher et al., 2017).

Microorganisms convert cellulose of straw into soluble sugars and glucose by producing cellulase (Wood and Garcia-Campayo, 1990), which is the typical example that turning the waste into treasure and largely increase the added value of the target agricultural product. Cellulase is an enzyme system consisting of a mixture of several enzymes, which are primarily classified into into three main types: β-glucosidase, exo-β-1,4-glucanases, and endo-β-1,4-glucosidases (Uzuner and Cekmecelioglu, 2019). All three types of enzymes belong to the glycoside hydrolases (GH) family as described in the Carbohydrate Activity Enzyme Database (CAZY), and these enzymes hydrolyze cellulose thoroughly in a manner of activity complementarity and coordination (Pollegioni et al., 2015). Cellulases are usually produced by microorganisms growing on humus-rich soils, and many plant pathogens can also express cellulases for their effective invade of plant cell wall (Lakshmi and Narasimha, 2012). Therefore, cellulase-producing microorganisms can be easily isolated from soil samples in forests and nature reserves (Lakshmi and Narasimha, 2012).

Bacteria and fungi in soil are the primary organisms responsible for degradation of cellulose which is the predominant polymeric component of plant cell walls (Baldrian and Valášková, 2008). Fungi have a relative higher capacity to produce cellulases than bacteria (Mondal et al., 2021), of which *Penicillium* sp. (Du et al., 2018), *Trichoderma* sp. (Dashtban et al., 2009), and *Aspergillus* sp.(Zhang et al., 2014) have been extensively studied in cellulases production. However, fungi pose challenges for transgenic manipulation, which hinders their practical application in cellulose degradation (Wang et al., 2013). Conversely, bacteria are often considered to be perfect tools for genome manipulation and functional modification due to their relative small genome size and with the ability to clone individual cellulases or express recombinant cellulases (Ma et al., 2020), as well as a shorter and more stable production cycle of bacterial cellulases. Hence, there is great potential for bacteria to develop stable cellulases in cellulose degradation (Ladeira et al., 2015). In recent years, numerous studies have been carried out to screen and identify bacteria with the ability to degrade cellulose from a variety of sources. For example, *Bacillus subtilis* BY4 (Kazeem et al., 2017) was isolated from oil palm empty fruits-chicken dung compost, *Bacillus sphaericus* BS-5 (Xu et al., 2017) was isolated from soil, and *Bacillus subtilis* BY-16 (Ma et al., 2015) was isolated from the gastrointestinal tract of Tibetan pig. However, the cellulose-degrading ability of *Rhodococcus* and *Pseudomonas* have not been reported so far, while the current library of bacteria with cellulose-degrading ability is not sufficient (Ma et al., 2020). Therefore, screening different bacterial species with cellulase degrading ability is crucial for the research and application of cellulose-degrading in straw’s further development and utilization on agriculture.

However, the low cellulase yield of bacteria has significantly limited the wide usage in practical applications, especially within industry settings. Enhancing the cellulase production yield of targeted bacterial strains through the optimization of fermentation conditions and genetic manipulation of crucial cellulase-encoding genes is essential for overcoming application barriers (Campano et al., 2015). To adjust the bacterial culture conditions, which mainly include temperature, pH, carbon source, and nitrogen source, the target bacteria could efficiently increase the yield and activity of the cellulase (Aswini et al., 2020). Recent advances in whole-genome sequencing and bioinformatics prediction technologies have identified series of gene candidates for cellulase (Yadav and Dubey, 2018; Yadav et al., 2019; Lu et al., 2020; Paudel et al., 2022; He et al., 2023), thereby providing a cellulase gene library for potential use in future application. According to the coding information, it’s possible to genetically improve the cellulase yield and single enzymatic activity of the target bacteria, which will be one of the most effective ways to lift the industrial application restrictions of the target bacteria in future. For instance, the cellulase gene from *Bacillus* was cloned and expressed efficiently in *E. coli*, and the corresponding cellulase has exhibited high catalytic activity *in vitro* (Kim and Ku, 2018). However, the entire set of cellulase systems in different microbial is not yet fully explored, and a deep understanding of the genomic information of different cellulase-producing strains is of great urgent and important for development of high yield and enzymatic activity cellulase-producing genetic engineered bacteria.

Our investigation provided the candidate strategy and the target bacterial strain from theoretical basis for the full utilization of straw resources on industry in the future.

## Materials and methods

### Isolation and screening of strains

After removing plant debris on ground surface, the soils (20 cm deep) were collected and placed in sterile sealed bags. Approximately 10 g of soil sample was added to 40 mL sterile distilled water in a 50 tube (Cat. No. CLS430304, Corning, Millipore, Sigma-Aldrich, Shanghai), followed by shaking 10 min for fully dissolving of the soil microorganisms. After allowing the tube to stand for 5 min, 1 mL of the supernatant was carefully extracted and spread onto plates containing enrichment CMC medium (CMC -Na 10 g/mL, KH_2_PO_4_ 1 g/mL, NaCl 0,1 g/mL, FeCl_3_ 0,01 g/L, NaNO_3_ 2,5 g/L, CaCl_2_.6H_2_O 0,1 g/L). These plates were then incubated in a constant temperature incubator (Cat. No. HD201801773, Boxun, Shanghai, China) at 28°C for 3 days. The bacteria obtained from the enrichment medium were diluted 10^−2^, 10^−3^, and 10^−4^ and coated evenly on above medium. The bacteria were then cultured at 28°C for a further 72 h to facilitate the development of pure colonies. Subsequently, the purified strains were inoculated in the Congo Red carboxymethyl cellulose medium and cultured at 28°C for 72 h, followed by preliminary screening based on the ratio of the diameter of the hydrolysis circle to the diameter of the colony.

### Molecular identification of bacteria

Single colonies were inoculated into LB liquid medium (NaCl 10 g/L, Yeast extract 5 g/L, Tryptone 10 g/L) and incubated at 28°C, 200 g on a shaker (Cat.No.MQ2023100755YA, Minquan, Shanghai, China) for 24 h. The bacterial culture medium was centrifuged at 10,000 g, and the supernatant was aspirated. The genomic DNA of the bacteria was extracted employing the Solarbio Bacterial Genomic DNA Extraction Kit (Cat. No. D1600-50, Solarbio, Beijing, China). This extracted genomic DNA served as a template for the polymerase chain reaction (PCR) amplification of the 16S rDNA fragments, using 27F (5’-AGAGTTTGATCCTGGCTCAG-3′) and 1492R (5’-GGCTACCTTGTTACGACTT-3′) as the forward and reverse primers, respectively. The PCR products were detected via 1% agarose gel electrophoresis and analyzed using a gel imaging system (JS-780D, Peiqing Technology, Shanghai, China) to identify the presence of target bands and then purified using agarose gel DNA purification Kit (Takara, Dalian, China). These bands were cut, recovered, and inserted into the pMD19-T vector. The ligated vector was transformed into *Escherichia coli* DH5α cells. A single colony was selected and sent to Shanghai Sangon Biologicals Inc. for sequencing analysis. The sequencing results were blasted in National Center for Biotechnology Information (NCBI). These sequences were then aligned using the MUSCLE function in MEGA 11 software, and a phylogenetic tree was constructed utilizing the Neighbor-Joining method with a bootstrap value of 1000. Finally, the phylogenetic tree was visually enhanced using ITOL for further analysis.

To accurately characterize strains YZ02 and YZ03, we constructed phylogenetic trees based on the common core genes of bacterial genomes of the same genus in the NCBI database. This process was facilitated by the EasyCGTree software platform, developed by Zhang et al., which operates on the Perl programming language (Zhang et al., 2023). Utilizing whole-genome sequencing data, we acquired the amino acid sequences for the majority of bacteria in the corresponding genus from the NCBI genomic database. A profile HMM (hidden Markov model) database was established using 120 ubiquitous genes (equating to 120 protein domains) present across the domain Bacteria (Parks et al., 2017). We then used HMMER^1^ to search for homologous genes, MUSCLE (Manuel, 2013) for sequence comparisons, trimAI (Capella-Gutiérrez et al., 2009) to screen conserved regions, and FasTree to construct a super tree (ST) based on the 120 core genes, the bacteria were identified to the species level.

### Scanning electron microscopy (SEM)

Bacteria were cultured in liquid LB medium for 24 h, followed by centrifugation at 8000 g for 1 min. The cells were then washed with 0.1 M PBS buffer solution (pH 7.4) and fixed in 2.5% glutaraldehyde at 4 degrees Celsius overnight. Subsequently, the bacteria underwent thorough washing with distilled water and PBS buffer. Dehydration was carried out using a series of alcohol solutions with increasing concentrations: 30, 50, 70, 80, 90, 95, and 100%. Each alcohol concentration was used twice, with each exposure lasting for 15 min. The samples were then dried in a critical point drier using CO_2_. For electron microscopy preparation, the dried specimens were coated with approximately 35 nm of gold–palladium (Jesus et al., 2015). The sample was observed and photographed under condition of a voltage of 5.00 kV and a magnification of 15,000-fold using a field emission scanning electron microscope (GeminiSEM 300, Carl Zeiss, Oberkochen, Germany).

### Cellulose activity assay

The enzymatic activities of various cellulase enzymes were determined by employing the 3,5-dinitrosalicylic acid (DNS) method, a colorimetric assay technique that is widely used to quantify reducing sugars, such as glucose, that result from enzymatic reactions involving carbohydrate substrates (Miller, 2002). This method was originally described by and involves the addition of DNS reagent to the reaction mixture containing the enzyme and its substrate. Upon heating, the reducing sugars react with the DNS reagent, resulting in a color change that can be measured spectrophotometrically. The absorbance correlates with the concentration of reducing sugars and, hence, the activity of the enzyme. Carboxymethylcellulose (CMC) was the substrate to assess the activity of endo-1,4-β-glucanase (CMCase), which breaks down the internal bonds of the cellulose molecule, resulting in shorter polysaccharide chains. Absorbent cotton, which is composed of long-chain cellulose fibers, was used to measure the activity of exo-1,4-β-glucanase (C1 enzyme), which cleaves cellulose from the ends of the chains to release cellobiose or glucose. Salicin, a glucoside derived from willow bark, served as the substrate for β-glucosidase (BG) (Somogyi, 1952), which hydrolyzes cellobiose and other β-d-glucosides to glucose. The enzyme activity unit is defined according to international regulations: the amount of enzyme required to catalyze the hydrolysis of cellulose to 1 μmol of glucose in 1 min is defined as one enzyme activity unit (IU/mL) (Eveleigh et al., 2009). To investigate the temporal dynamics of enzyme activity, samples were collected at 12-h intervals, centrifuged, and the supernatants were preserved at −20 Celsius. This systematic method facilitated the preservation of enzymatic activity and the exploration of the relationship between enzyme production and time elapsed. Fermentation cultures of the isolates were performed using straw powder as the sole carbon source. Each 10 mL test tube contained 0,2 g (W_0_) of straw powder. After 15 days of fermentation, the remaining straw powder in the test tubes was dried and weighed (W_15_). The straw degradation rate was calculated as (W_0_ - W_15_) / W_0_.

### Gene prediction and functional annotation

The genomic DNA extraction from bacterial tissue utilized an optimized SDS (Sodium Dodecyl Sulfate) method, which is effective in lysing cells and denaturing proteins, making it a popular choice for extracting high-quality DNA. Specifically, 1,0 g of bacterial tissue was processed to extract DNA. Samples were then tested and purified using Ligation Sequencing Kit (SQK-LSK110, Oxford Nanopore Technologies, Oxford, UK). Libraries were created according to the instructions. For the preparation of small fragment libraries, the VAHTS Universal Plus DNA Library Prep Kit (ND617-01, Vazyme, Nanjing, China) and MGI V2/for Illumina V2 Kit (NDM627-01, Vazyme, Nanjing, China) were employed. After passing quality control, the libraries were sequenced using the Nanopore PromethION and Illumina NovaSeq 6,000 platforms. After sequencing, data processing involved filtering splices, short fragments, and low-quality reads to retain high-quality data for further analysis. The bacterial genome assembly was initiated with Nanopore long-read data using Flye software, an assembler designed for long reads that can efficiently manage the complexities and errors associated with them. The initial assembly was then refined using Pilon software (1.24) with Illumina short-read data for error correction, ensuring a high-quality genome assembly (Wang et al., 2014). Gene sequences extracted from the assembled genome were annotated against functional databases such as COG, KEGG, Uniprot, and Refseq using BLAST+ (version 2.11.0+), a widely used tool for comparing nucleotide or protein sequences to sequence databases and identifying functional information. Additionally, to specifically identify carbohydrate-related enzyme genes, protein sequences were annotated using HMMER against the CAZy database, a specialized resource for carbohydrate-active enzymes.

## Result

### Isolation and morphology observation of these cellulose-degrading bacteria

**Figure 1:**
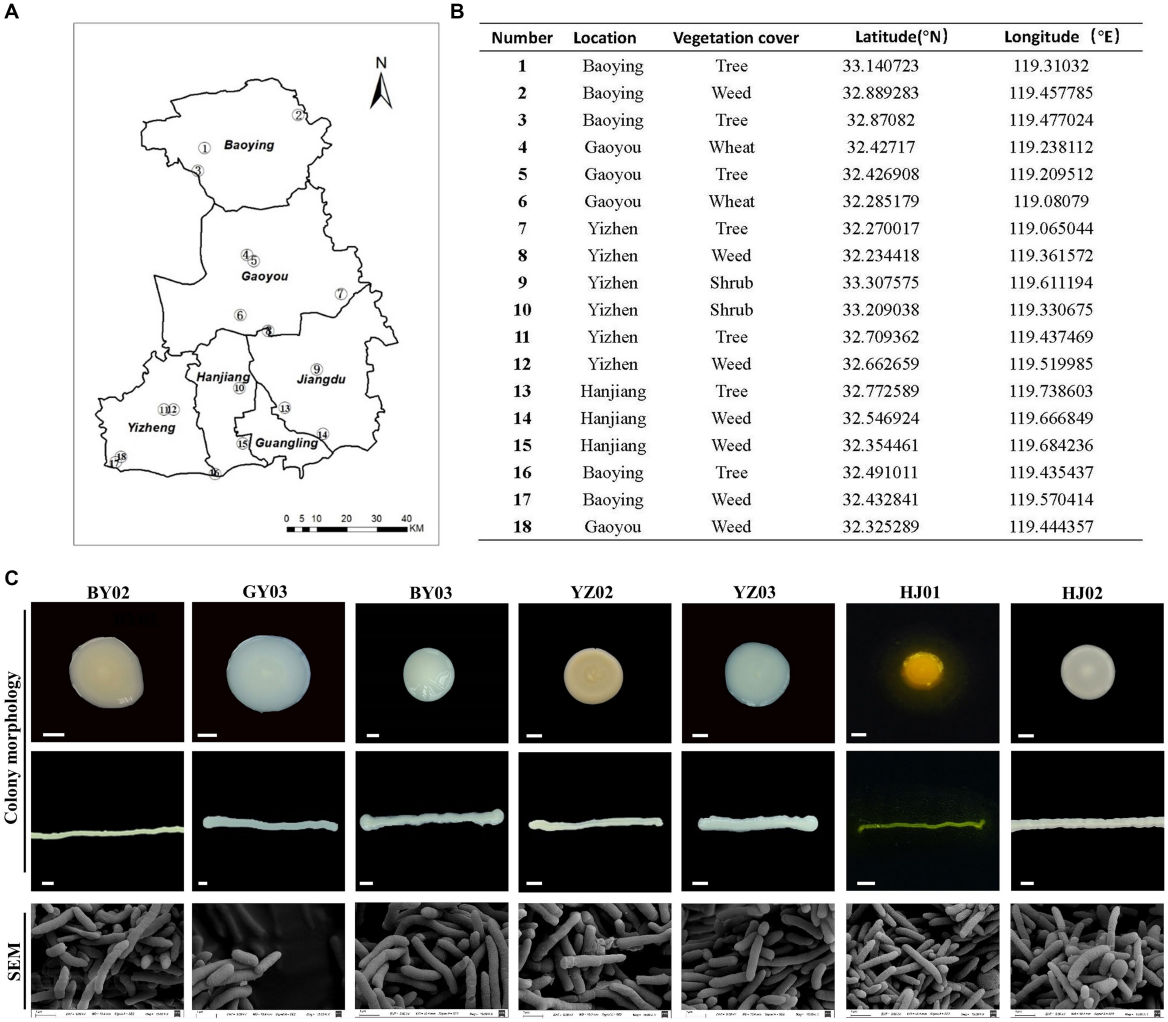
Isolation and Screening of Cellulose-Degrading strains from soil. **(A)** Sampling sites of the collected soil samples. **(B)** Detailed information on each sampling location. **(C)** Representative images of isolated colonies on selective media plates and under scanning electron microscopy (SEM) images of the same isolates. Baoying-02, the number 2# isolated from Baoying city, so named it BY02, it is *Pseudomonas mosselii*; the number 3# Gaoyou-03 isolated from Gaoyou city, so named it GY03, it is *Pseudomonas putia*; Baoying-03, the number 3# isolated from Baoying city so name it BY03, it is *Pseudomonas glycinae*; Yizheng-02, the number 3# isolated from Yizheng city so name it YZ02, it is *Rhodococcus wratislaviensis*; Yizheng-03, the number 3# isolated from Yizheng city so name it YZ03, it is *Pseudomonas xanthosomatis*; Hanjiang-01, the number 1# isolated from Hanjiang district so named it HJ01, it is *Pseudomonas straminea*; Hanjiang-02, the number 2# isolated from Hanjiang so named it HJ02, it is *Prestia qingshengii*; Scale bar: 0.3 mm.

Soil samples were collected from various humus-rich and undisturbed soil environment including parks, protected areas, farmlands, and wastelands in Yangzhou City (Figures 1A,B). Single colonies obtained via the dilution plating method were subsequently examined for colony morphology, size, and the color of metabolites on the plate. Scanning electron microscopy (SEM) analysis was employed to examine each bacterial strain’s morphology. It was found that all of the strains are rigid rod-shaped particle (Figure 1C), which implied the species of these bacterial.

After 48 h of culture, the YZ02 strain’s colony exhibited a slightly red color with a lightened and opaque surface. SEM observation revealed that YZ02’s cells were rod-shaped (Figure 1C). The YZ03 strain’s colony was round with a smooth surface and white color; its cells also displayed a rigid rod shape under SEM (Figure 1C). The HJ01 strain’s colony was yellow, emitting yellow fluorescence around the colony, with rod-shaped cells under SEM (Figure 1C). The colony of HJ02 strain were round, white, and opaque, and their cells were also rod-shaped (Figure 1C). The colony of BY03 strain were 3–5 mm in diameter and showed yellowish color, and their cells were also rod-shaped (Figure 1C). The colony of GY03 strain showed milky white color and was opaque, and the cells are also rod-shaped. The colonies of strain BY02 appeared as round, beige, opaque structures with a raised center, regular, smooth, and moist edges, and contained rod-shaped cells. All the described strains were categorized as rod-shaped bacteria, yet precise molecular identification is necessary to determine their taxonomic classifications and species identities conclusively.

### Molecular identification of the obtained bacteria’s strain

**Figure 2:**
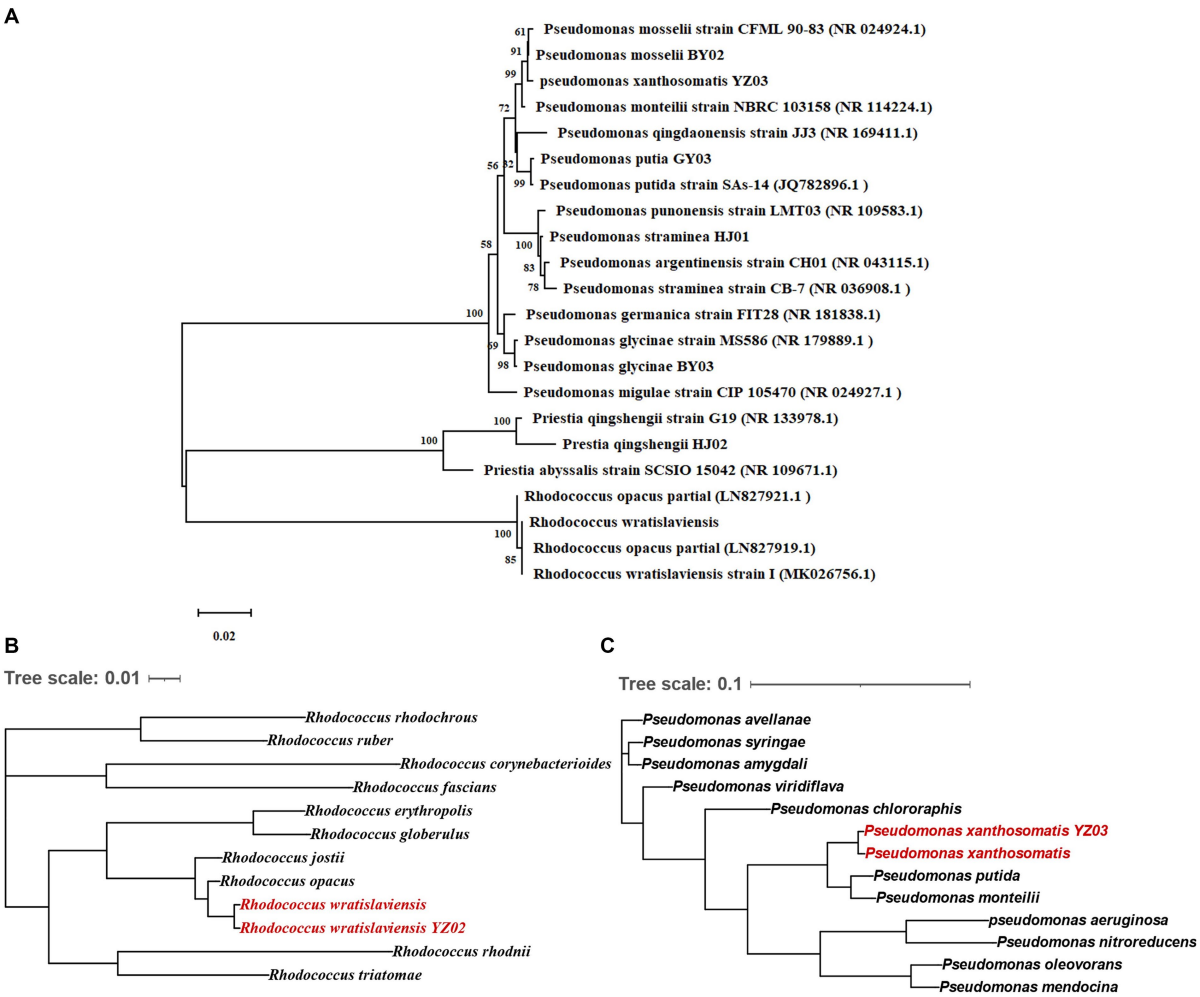
Neighbor-joining tree showing the phylogenetic position of cellulose-degrading bacteria. **(A)** Neighbor-joining tree constructed based on 16S rRNA gene sequences depicting the genetic relatedness of strains YZ02, GY03, BY03, YZ02, YZ03, HJ01 and HJ02. The evolutionary history was inferred using the Neighbor-Joining method. The optimal tree is shown. The percentage of replicate trees in which the associated taxa clustered together in the bootstrap test (1,000 replicates) are shown next to the branches The evolutionary distances were computed using the Maximum Composite Likelihood method. **(B)** This tree illustrates the evolutionary relationships of strain YZ02 based on the sequence alignment of a panel of 120 conserved genes. Each node represents a distinct bacterial taxon, and the branches reflect the inferred evolutionary pathways and genetic distances between species. **(C)** This tree illustrates the evolutionary relationships of strain YZ03 based on the sequence alignment of a panel of 120 conserved genes. Each node represents a distinct bacterial taxon, and the branches reflect the inferred evolutionary pathways and genetic distances between species.

For further identification of the isolated bacterial strains, 16S rRNA gene sequencing was conducted with specific primer pairs. The PCR amplification yielded fragments of 1,500 bp, indicative of the 16S rRNA gene presence in the strains. Sequences were submitted to NCBI for BLAST analysis, and a phylogenetic tree was constructed based on the 16S rRNA gene sequences (Figure 2A). Phylogenetic analysis revealed that the seven strains belong to three genera: *Rhodococcus, Pseudomonas*, and *Priestia*. *R. wratislaviensis* YZ02 shared a close phylogenetic relationship with *Rhodococcus wratislaviensis* (NR 026524.1) (Figure 2A), while strain HJ02 aligned closely with *Priestia qingshengii* strain G19 (NR 133978.1) (Figure 2A). The remaining strains BY02, YZ03, GY03, HJ01 and BY03 were classified as *Pseudomonas*, exhibiting close relationships with *Pseudomonas mosselii* strain CFML 90–83 (NR 024924.1), *Pseudomonas xantholysini*, *Pseudomonas putida* strain Sas-14 (JQ782896.1), *Pseudomonas straminea* strain CB-7 (NR 036908.1), and *Pseudomonas glycinae* MS586 (NR 179889.1), respectively (Figure 2A). Supplementary Table S1 provides the detailed information of each isolate.

In conjunction with prior morphological and SEM observations (Figure 1C), we confirmed that strain HJ02 is *Priestia qingshengii*, aligning with characteristics of *Priestia qingshengii* previously isolated from a rock surface (Xi et al., 2014). Similarly, strain BY03 was identified as *Pseudomonas glycinae,* consistent with previous isolations from soybean roots (Jia et al., 2020). The morphological characteristics of strain HJ01 were consistent with those described by Iizuka and Komagata (1963). Nevertheless, due to the inherent limitations of 16S rRNA gene resolution at the species level, *R. wratislaviensis* YZ02 and *P. xanthosomatis* YZ03 were selected for whole-genome sequencing to provide more definitive identifications.

Upon obtaining the whole-genome maps for these strains, a comparative genomic analysis with other strains within the same genus was conducted to construct a phylogenetic tree based on 120 core genes (Figures 2B,C). The analysis confirmed that *R. wratislaviensis* YZ02 and *P. xanthosomatis* YZ03 exhibit the closest genetic affiliations to *Rhodococcus wratislaviensis* and *Pseudomonas xanthosomatis*, respectively. Therefore, we definitively categorized strain YZ02 and strain YZ03 as *Rhodococcus wratislaviensis* YZ02 and *Pseudomonas xanthosomatis* YZ03, respectively.

### Cellulase enzyme activities assay

**Figure 3:**
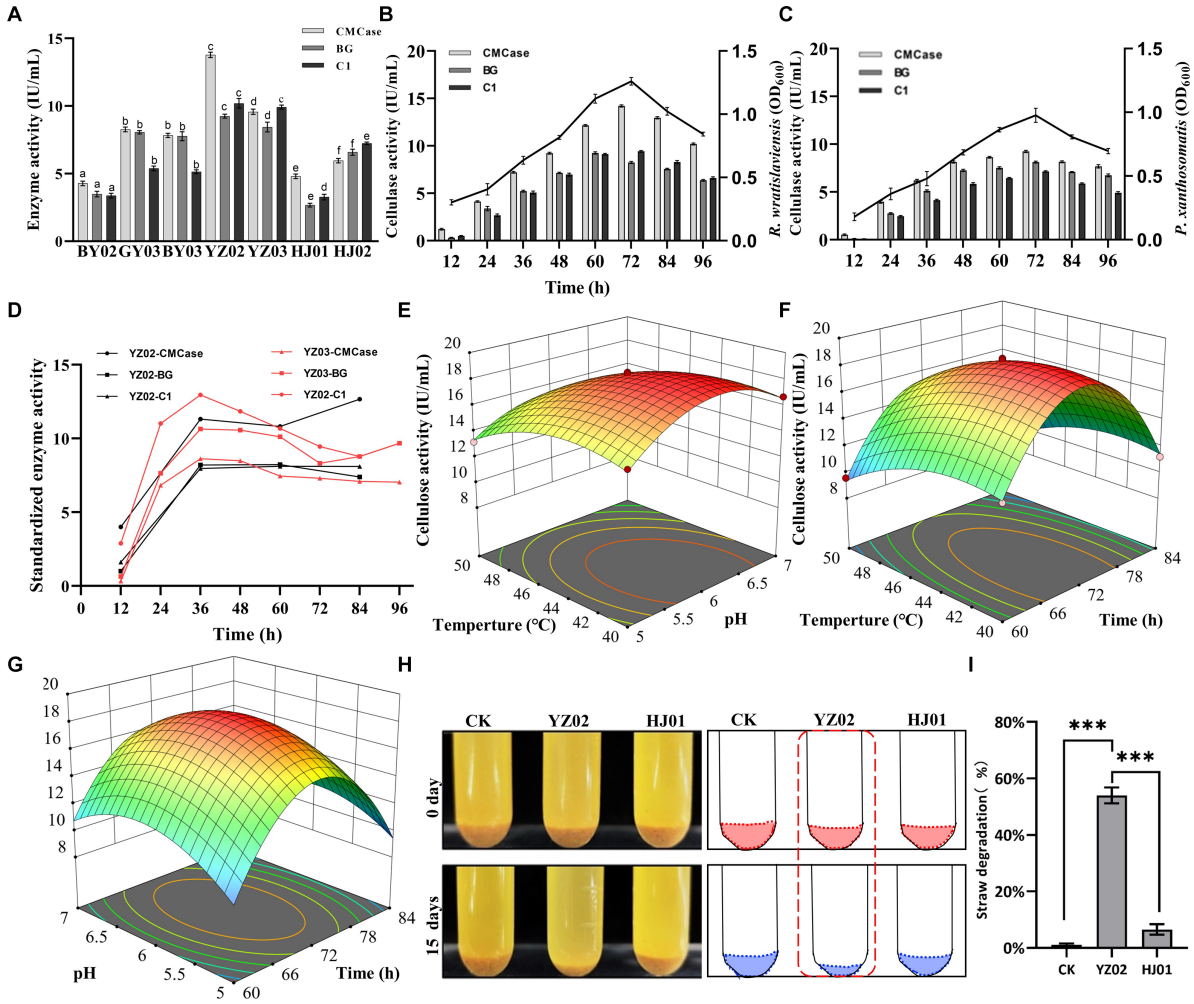
Determination of cellulase activity of the isolated strains. **(A)** CMCase, BG, and C1 enzymes represent endo-1,4-β-glucanase, β-glucosidase, and endoglucanase, respectively, using CMC, salicin, and absorbent cotton as reaction substrates Enzyme activity was measured at 12 h intervals. The values represent the means of three biological replicates, and the error bars indicate the standard error of the mean. The cellulase activity of each isolate was monitored over a 72 h fermentation period using the 3,5-dinitrosalicylic acid (DNS) method. Different letters indicate significant differences between groups (*p* < 0.05) **(B)** Trend of CMCase, BG, and C1 enzyme activities of strain YZ02 over time. The left Y-axis represents enzyme activity, and the right Y-axis represents the bacterial OD600 value. **(C)** Trend of CMCase, BG, and C1 enzyme activities of strain YZ03 over time. The left Y-axis represents enzyme activity, and the right Y-axis represents the bacterial OD600 value. **(D)** The CMCase, BG, and C1 enzyme activity data of strains YZ02 and YZ03 were normalized. The normalized enzyme activity data were obtained by dividing the cellulase activity by the bacterial OD600.The Y-axis represents the relative cellulase activity. **(E)** Response surface diagram of temperature and pH effect on the CMCase activity of strain YZ02. **(F)** Response surface diagram of temperature and time effect on the CMCase activity of strain YZ02. **(G)** Response surface diagram of Ph and time effect on the CMCase activity of strain YZ02.To assess the straw powder degradation capability of *R. wratislaviensis* YZ02, the fermentation medium devoid of bacteria served as a blank control, while the degradation efficacy of strain HJ01 acted as a positive control. **(H)** Using straw powder as a carbon source, inoculated with cellulolytic bacteria and fermented for 15 days. **(I)** The degradation rate of straw powder by strain YZ02 and strain HJ01 was obtained by subtracting the weight of the original straw powder in the test tube from the weight of the straw powder remaining after 15 days of fermentation and dividing by the weight of the original straw powder.

To investigate the cellulase activities of the isolated strains and identify the one with the highest activity, we employed the DNS (3,5-dinitrosalicylic acid) assay to measure the concentration of reducing sugars. This approach facilitated the precise and accurate assessment of the strains’ cellulase activities. As shown in Figure 3A, after 72 h of fermentation, all seven strains exhibited satisfactory enzyme activities. However, *R. wratislaviensis* YZ02 and *P. xanthosomatis* YZ03 stood out, exhibiting the most robust CMCase, endoglucanase (C1), and β-glucosidase (BG) activities compared to the others. Consequently, *R. wratislaviensis* YZ02 and *P. xanthosomatis* YZ03 were chosen for subsequent investigations to explore their enzyme production peaks. For *R. wratislaviensis* YZ02, the activities of CMCase and C1 peaked at 72 h of fermentation, while the activity of BG reached its maximum at 60 h. Following this peak, the activities of all cellulases gradually declined. To explore the relationship between cellulase activity and bacterial growth, we quantity the cells at OD600 at various time points to indicate the bacterial number. The results indicated that the bacterial count exhibited a similar trend to that of cellulase activities (Figure 3B). Similarly, for *P. xanthosomatis*, the activities of all three cellulases peaked at 72 h of fermentation, and the OD600 values mirrored the trend of cellulase activities (Figure 3C). After standardizing the data, we observed that the relative maximum enzyme activity of YZ03 (12.95 IU/mL) exceeded that of YZ02 (12.66 IU/mL) (Figure 3D). Subsequently, response surface methodology (RSM) experiments were conducted to investigate the optimal fermentation conditions for YZ02. The results indicated that under the conditions of a fermentation time of 72 h, pH of 6, and temperature of 45°C, *R. wratislaviensis YZ02* achieved the highest cellulase activity (Figures 3D–F). Figures 3G,H demonstrate that after 15 days of fermentation, 55% of the straw powder in the medium was degraded and *R. wratislaviensis* YZ02 had a strong ability to degrade straw powder. Figure 3G demonstrates that *R. wratislaviensis* YZ02 exhibits strong degradation capability towards sieved straw powder. After 15 days of fermentation, the treatment group with the addition of *R. wratislaviensis* YZ02 had only half of the straw powder remaining (Figure 3G). The degradation capability of *R. wratislaviensis* YZ02 was significantly higher compared to strain HJ01. Figure 3I quantitatively analyzed the residual straw powder in each treatment group by weighing, revealing that *R. wratislaviensis* YZ02 degraded 55% of the straw powder in the system after 15 days of fermentation.

### Whole genome sequencing genome sequencing and annotation

**Figure 4:**
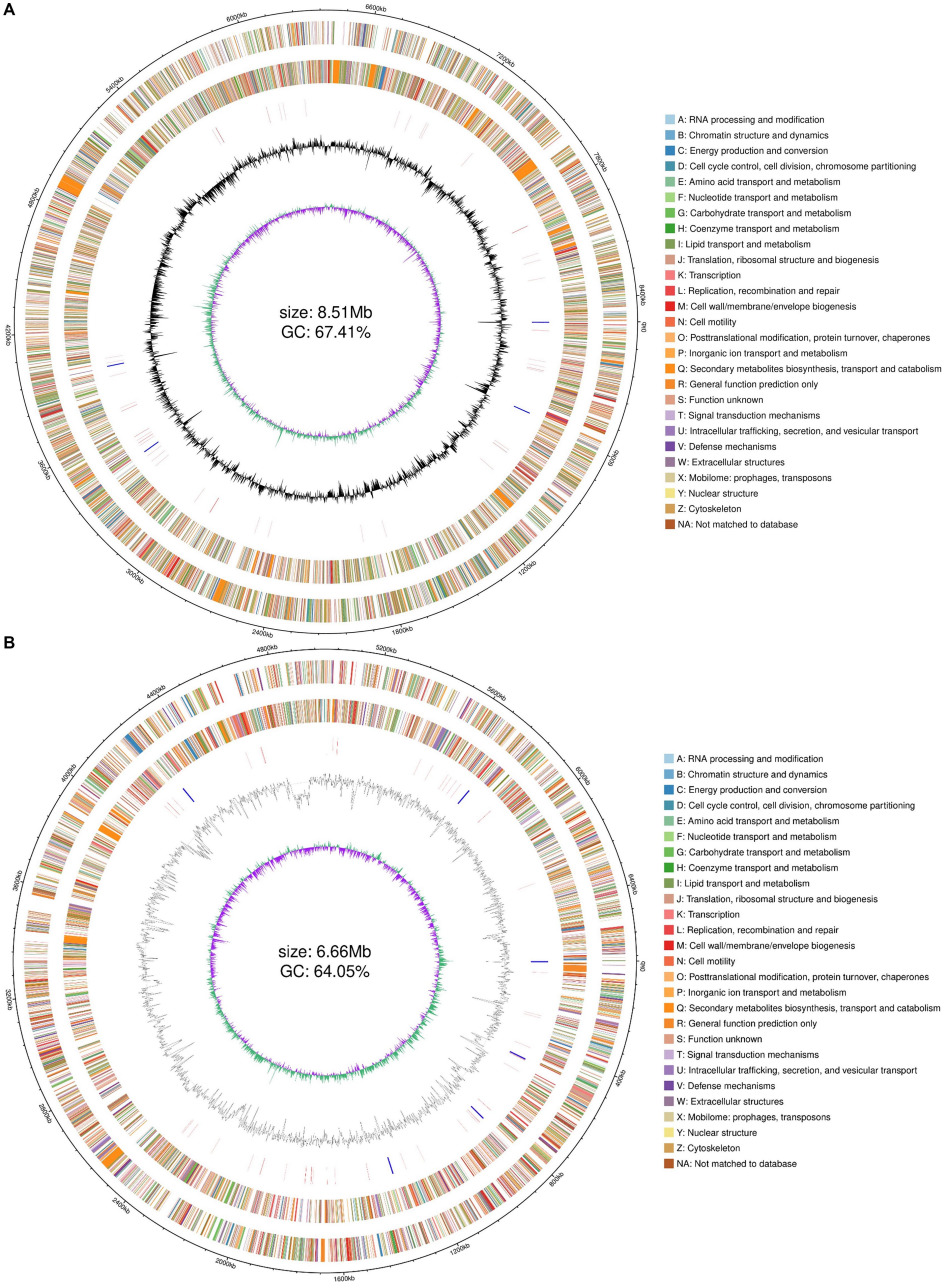
Whole-genome visualization map of *Rhodococcus wratislaviensis* YZ02 and *Pseudomonas xanthosomatis* YZ03. From the outer circle to the inner circle, ring 1, genomic coordinates; ring 2, genes on the positive strand of the genomic sequence, with different colors representing different COG functional classifications; ring 3, genes on the negative strand of the genomic sequence, with different colors representing different COG functional classifications; ring 4, rRNAs and tRNAs on the genomic sequence, with the rRNAs in blue and the tRNAs in red; ring 5, GC content of the genomic sequence; ring 6, GC skew profile of the genomic sequence.

Combined with the results of previous enzyme activity analyses, we selected two strains YZ02 and YZ03, with the highest cellulase activity from the isolates for Whole genome sequencing and annotation. Whole genome sequencing of *R. wratislaviensis YZ02* and *P. xanthosomatis YZ03* was performed based on the Nanopore triple sequencing technology platform and the second-generation sequencing technology platform. The genome size of *R. wratislaviensis* YZ02 was 8.51 Mb, encompasses 8,466 genes, including 4 23S rRNAs, 4 16S rRNAs, and 4 5S rRNAs, of these, 3,776, 8,308, 6,253, 7,080, and 6,975 genes were, respectively, annotated in the KEGG, Nr, GO, COG, and Pfam databases (Supplementary Table S2). The genome of *P. xanthosomatis*, sized at 6.66 Mb, comprises 5,745 genes with 6 23S rRNAs, 6 16S rRNAs, and 7 5S rRNAs. These were annotated in the KEGG, Nr, GO, COG, and Pfam databases, with annotations numbering 3,385, 5,703, 3,940, 4,758, and 4,830, respectively (Supplementary Table S2). Genomic data, including genome structure annotations, GC content, and COG functional annotations, were synthesized using an R package to depict the genomic circle (Figures 4A,B). The results demonstrated that *R. wratislaviensis* YZ02 exhibited a genome size of 8.51 Mb and a GC content of 67.41% (Figure 4A), while *P. xanthosomatis* exhibited a genome size of 6.66 Mb and a GC content of 64.05% (Figure 4B), and clearly showed the relationship between genome components and genome positions.

### Homologous gene clusters involved in glucose metabolism in genome annotation

**Figure 5:**
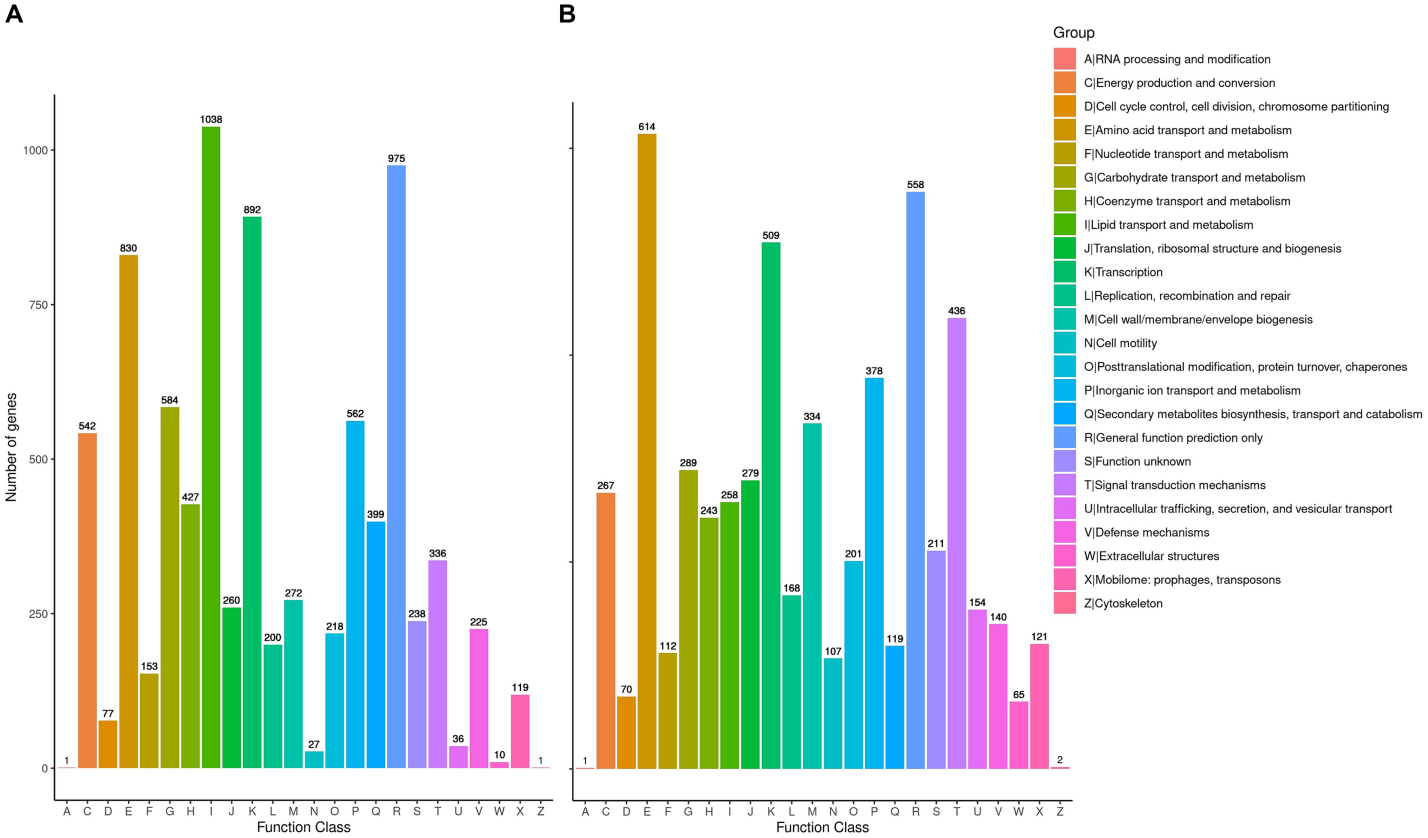
Clusters of Orthologous Groups annotation analysis. **(A,B)** It illustrates the distribution of genes within the genomes of strains YZ02 and YZ03, respectively, according to their predicted functional categories. Vertical axis is the number of genes annotated under COG classification while the horizontal axis is the content of each COG classification.

To delve deeper into the cellulolytic capabilities of *R. wratislaviensis* YZ02 and *P. xanthosomatis* YZ03at the genetic level, these strains were annotated in the COG database, revealing 7,080 and 4,758 genes, respectively (Figures 5A,B). These annotations facilitated an analysis of genes associated with carbohydrate metabolism. For amino acid transport and metabolism, *R. wratislaviensis* YZ02 and *P. xanthosomatis* YZ03 possessed 830 (11.72%) and 614 (12.91%) genes, respectively. In terms of carbohydrate transport and metabolism, the gene counts were 584 (8.25%) for *R. wratislaviensis* YZ02 and 289 (6.07%) for *P. xanthosomatis* YZ03. Among the identified genes, strains YZ02 harbor COG2723 (β-glucosidase), COG3507 (beta-xylosidase), and COG1427 (exoglucanase) (Figure 5A). *P. xanthosomatis* possessed COG1427 (exoglucanase) (Figure 5B). The presence of a considerable number of genes related to carbohydrate metabolism and transport indicates that and *P. xanthosomatis* YZ03 possess the genetic basis for cellulose degradation.

### KEGG and go database annotations

**Figure 6:**
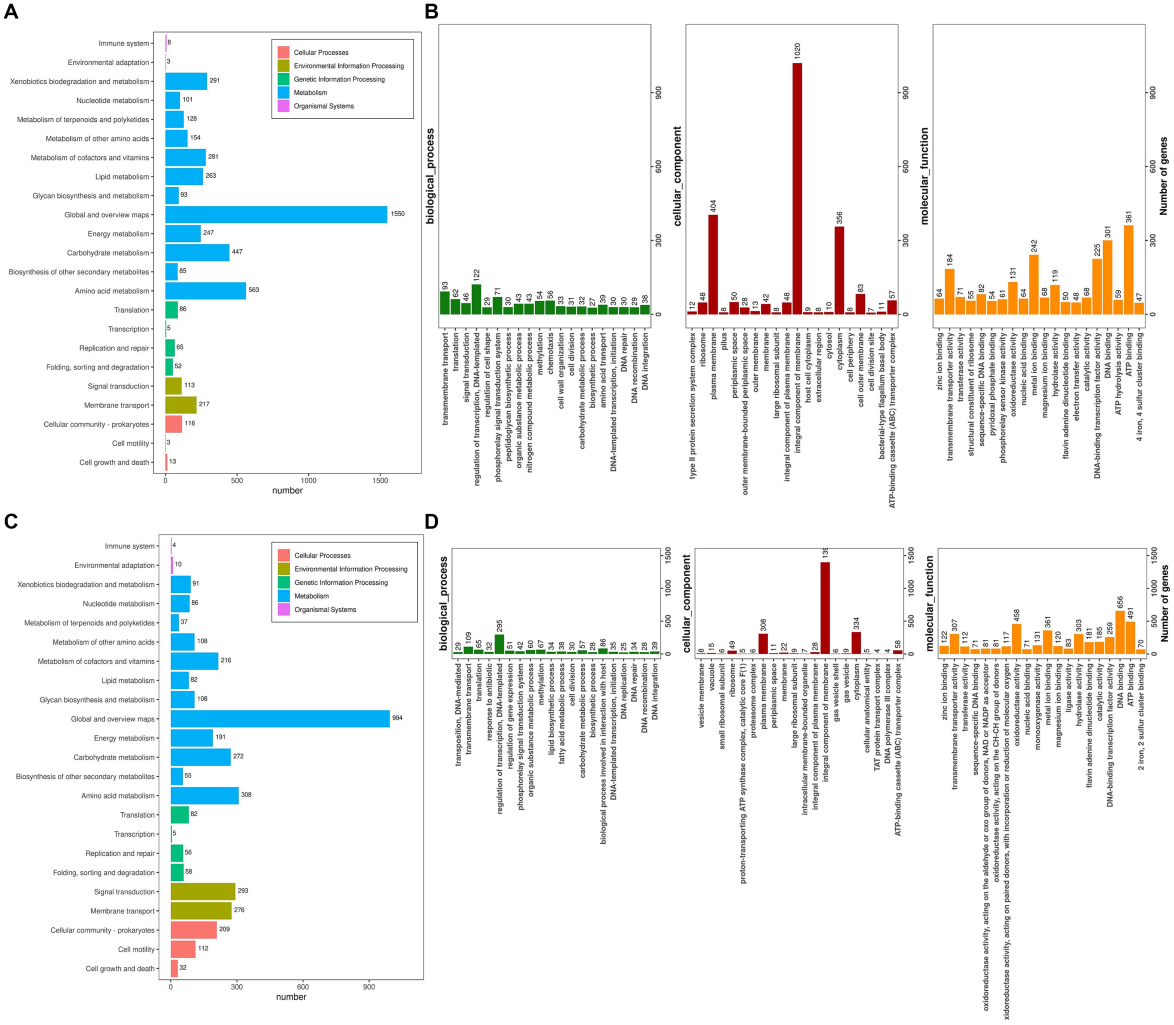
KEGG pathway analysis and Gene ontology cluster analysis of *Rhodococcus wratislaviensis* YZ02 and *Pseudomonas xanthosomatis* YZ03. **(A)** The KEGG annotation analysis of *R. wratislaviensis* YZ02and **(C)** The KEGG annotation analysis of *P. xanthosomatis* YZ03 The horizontal axis is the number of genes annotated under KEGG pathway classification. The vertical axis is KEGG pathway classification, and different colors represent different classifications. **(B)** The GO annotation analysis of *R. wratislaviensis* YZ02. **(D)** The GO annotation analysis of *P. xanthosomatis*. The horizontal axis generally represents the count or proportion of genes within each GO term, showing the extent to which certain functions or processes are represented in the organism’s genome. The vertical axis lists the GO terms that have been assigned to the genes. This axis would be divided into the main GO domains (Biological Process, Molecular Function, and Cellular Component), each potentially further subdivided into more specific terms.

The Kyoto Encyclopedia of Genes and Genomes (KEGG) serves as a pivotal database for the comprehensive annotation of enzymes catalyzing reactions across diverse metabolic stages, facilitating an in-depth understanding of enzyme roles within metabolic pathways during bacterial cellulose degradation. The genomes of *R. wratislaviensis* YZ02 and *P. xanthosomatis* YZ03 were annotated utilizing KEGG and subsequently categorized based on their involvement in specific KEGG metabolic pathways. KEGG pathway analyses uncovered notable disparities in gene involvement across 15 metabolic pathways between strains YZ02 and YZ03, particularly within carbohydrate pathways, enumerating 447 and 272 genes, respectively, (Figures 6A,C). Moreover, the glycolysis/gluconeogenesis pathway involved 53 and 34 genes in *R. wratislaviensis* YZ02 and *P. xanthosomatis* YZ03, respectively (Figures 6A,C).

Gene Ontology (GO) annotation results indicated that genes within *R. wratislaviensis* YZ02 and *P. xanthosomatis* YZ03 were predominantly enriched in molecular functions, while cellular components and biological processes were less represented (Figures 6B,D). Within biological processes, *R. wratislaviensis* YZ02 and *P. xanthosomatis* YZ03 harbored 295 and 122 genes, respectively, associated with transcription regulation. Furthermore, analysis revealed 656 and 301 genes related to DNA binding and 491 and 361 genes associated with ATP binding in strains YZ02 and YZ03, respectively (Figures 6B,D). Subsequent analysis of genes involved in carbohydrate metabolism and transport yielded 57 and 32 carbohydrate metabolism-related genes in strains YZ02 and YZ03, respectively, encompassing GO terms such as GO:0005975 (carbohydrate metabolic process), GO:0030246 (carbohydrate binding), and GO:0008643 (carbohydrate transport). These findings suggest that the abundance of carbohydrate metabolism-related genes in *R. wratislaviensis* YZ02 and *P. xanthosomatis* YZ03 underpins their cellulolytic capabilities.

### CAZyme annotations

**Figure 7:**
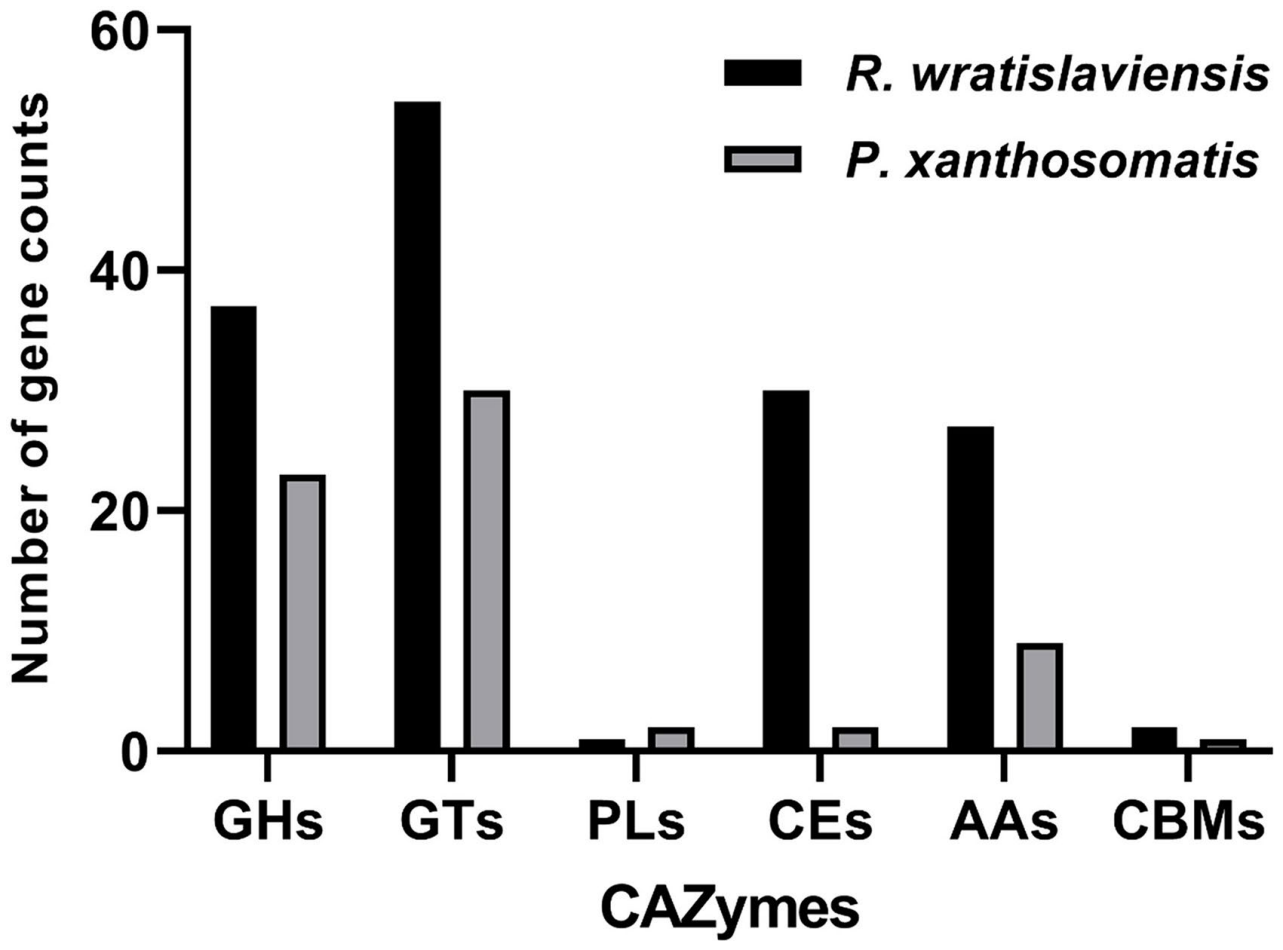
Carbohydrate-active enzymes family distribution in *Rhodococcus wratislaviensis* YZ02 and *Pseudomonas xanthosomatis* YZ03. The vertical axis (quantity of genes) represents the number of genes associated with each CAZyme family. A higher count indicates a greater potential capacity of the organism to engage in certain types of carbohydrate metabolism. The horizontal axis (types of genes/CAZyme families) categorizes the genes according to the specific CAZyme family they belong to, such as GH, GT, PL, CE, AA, and CBM.

Figure 7 illustrates the statistics of carbohydrate enzyme-related genes in strains YZ02 and YZ03, as annotated in the CAZy database. The glycoside hydrolase (GH) family and glycosyltransferase (GT) family account for the largest proportions. In *R. wratislaviensis* YZ02, there were 37 GH family genes and 54 GT family genes, whereas *P. xanthosomatis* YZ03 harbored 23 GH family genes and 30 GT family genes. Moreover, for *R. wratislaviensis* YZ02 and *P. xanthosomatis* YZ03 respectively, 30 and 2 CE genes, 1 and 2 PL genes, and 27 and 9 AA genes were identified (Figure 7). The genome of *R. wratislaviensis* YZ02 was found to contain 37 annotated cellulase-related genes, including 25 genes of the GH family. (Figure 7). These genes encode proteins such as β-glucosidase, which mainly belong to the GH1, GH2, GH3, and GH16 families, and endoglucanase, which mainly belongs to the GH5 family (EC 3.2.1.4). Furthermore, proteins encoded by the glucan-1,4-α- glucosidase (EC 3.2.1.3) gene mainly belongs to the GH15 family, and proteins encoded by the α-glucosidase (EC 3.2.1.20) gene mainly belong to the GH13, GH63, and GH76 families. *P. xanthosomatis’s* genome was found to possess 27 annotated cellulose-related genes, including those from the GH3, GH13, and GH15 families, which are pivotal in cellulose degradation. The presence of these genes enables bacteria to decompose cellulose efficiently and also responds to the ability of *R. wratislaviensis* YZ02 and *P. xanthosomatis* YZ03 to degrade cellulose at the genetic level.

## Discussion

Cellulose enzymes from bacteria are considered promising candidates for the conversion of lignocellulosic biomasses into fermentable sugars. Soil bacteria can produce various hydrolytic enzymes that play a crucial role in cellulose degradation (Hentges and Smith, 2018). In recent years a number of microorganisms with cellulose-degrading potential have been isolated from the soil, *B. subtilis K1*, isolated from soil, exhibits significant cellulose-degrading activity under cold conditions(He et al., 2023). Similarly, *Raoultella* sp. S12 (Bao, 2016) is known to degrade lignocellulose, and *Xanthomonas translucens*, isolated from rice straw, can be used in fuel cells (Khoirunnisa et al., 2020). *Bosea* sp. FBZP-16 (Houfani et al., 2017), *Paenibacillus lautus* strain BHU3 (Yadav and Dubey, 2018)*, Cellulomonas gilvus* sp. (Christopherson et al., 2013), these strains are all considered excellent cellulase-producing bacteria. However, a significant gap in the literature exists regarding the cellulose-degrading capabilities of *Rhodococcus wratislaviensis* and Pseudomonas xanthosomatis. We tested the enzymatic activities of the three most critical cellulases in cellulose degradation using carboxymethyl cellulose CMC, salicin, and cotton as substrates, and for the first time, discovered that *Rhodococcus wratislaviensis* and *Pseudomonas xanthosomatis* exhibit excellent cellulose-degrading abilities (Figure 3A). Additionally, we identified several rarely reported cellulose-degrading bacteria, including *Pseudomonas mosselii*, *Pseudomonas putia*, *Pseudomonas straminea*, and *Priestia qingshengii* as potential cellulase-producing strains (Figures 1C, 3A). These newly discovered cellulolytic bacteria expand the known range of cellulose-degrading bacteria and open up new avenues for industrial applications.

The cellulase activities of both strains YZ02 and YZ03 peaked after 60 to 72 h of fermentation, significantly surpassing those reported for several other cellulose-degrading bacteria. (Figures 3B,C). For instance, *Bacillus cereus*, as reported by Nema et al. (2015), exhibited a maximum cellulase activity of 0.213 IU/mL, and *Bacillus megaterium* isolated by Bhagat and Kokitkar (2021). Interestingly, we observed that the variations in cellulase activity closely mirrored the dynamic changes in bacterial cell numbers, elucidating the initial increase followed by a subsequent decrease in cellulase activity of the strains (Figures 3B,C). After standardizing the data to account for bacterial cell numbers, we observed an initial increase in cellulase activity, followed by a decrease and then a subsequent increase. This pattern is likely due to the accumulation and adsorption of reaction products inhibiting enzyme activity after it reaches its peak. Over time, the enzymes may desorb from the cellulose surface, alleviating the “clogging” effect and partially restoring their activity.(Andric et al., 2010). These findings not only demonstrate the high cellulase activity of strain YZ02 and YZ03 but also provide insights into the enzymatic dynamics during cellulose degradation, which can inform future industrial applications. Furthermore, we investigated the factors influencing the cellulase activity of *R. wratislaviensis* YZ02. Our results indicated that the highest cellulase activity was observed at a pH of 6 and a reaction temperature of 45°C (Figures 3D–F). This suggests that the cellulase from *R. wratislaviensis* YZ02 exhibits thermal stability and retains high activity across a broad pH range. Such characteristics are advantageous for industrial applications, as they have the potential to significantly reduce production costs. Moreover, there are very few studies that use rice straw powder as carbon source to investigate the utilization of straw cellulose by these strains. Our results demonstrate that *Rhodococcus wratislaviensis* can effectively degrade rice straw powders (Figures 3G,H). Rice straw, being a cheap and abundant cellulose substrate, has become a key raw material for cellulase production due to its high cellulose content and wide availability (Goodman, 2020). *Rhodococcus wratislaviensis* YZ02 may be highly effective in processing waste straw from Yangzhou, thereby enabling the recycling and utilization of agricultural waste. The ability of *R. wratislaviensis* YZ02 to process waste straw from Yangzhou highlights its potential for agricultural waste recycling, further underlining the practical implications of our findings.

The composition of fungal cellulase is less complex than that of bacteria, which can produce large amounts of cellulase extracellularly (Sukumaran et al., 2005). Among various fungi, *Aspergillus mushrooms* have higher cellulase activity compared to other genera. Although fungi have high enzyme-producing activity, bacteria is also efficient in cellulose degradation, with a wide range of cellulose-degrading enzymes and a strong organic matter degradation capacity. For example, Auffret et al. showed through transcriptomic analysis that *Rhodococcus wratislaviensis* can participate in the degradation of hydrocarbons, gasoline, and diesel fuel (Auffret et al., 2014), and Suresh *Rhodococcus Subashchandrabose* et al. showed that *R. wratislaviensis* is capable of degrading p-nitrophenol (Subashchandrabose et al., 2018), and Paola Grenni isolated *Rhodococcus* strains capable of degrading S-triazine from groundwater (Grenni et al., 2009). Compared with fungi, bacteria have the advantages of rapid growth, high application potential, short generation time and low cost for cellulase production (Westers et al., 2004), so cellulose conversion by bacteria is a highly efficient production strategy. Currently, *Fibrosomonas aeruginosa* and *Thermomonas brownii* have been widely studied and used for cellulase production.

Through whole-genome sequencing and analysis, we improved our understanding of the genomes and cellulose degradation pathways of cellulose-degrading bacteria. This knowledge provides a theoretical basis for the future engineering of *R. wratislaviensis* YZ02 and *P. xanthosomatis* YZ03 into cellulose-degrading microorganisms. Therefore, we selected *Rhodococcus wratislaviensis* YZ02 and *Pseudomonas Xanthosomatis* YZ03 for whole genome sequencing and analysis to obtain valuable genetic information. Through annotation in the COG, KEGG and GO database, it was found that *R. wratislaviensis* YZ02 and *P. xanthosomatis* YZ03 are equipped with a large number of genes essential for the metabolism of carbohydrates and other nutrients (Figures 5–7). Notably, *R. wratislaviensis* YZ02 had a significantly higher number of genes for metabolizing carbohydrates, starch, and sucrose compared to *P. xanthosomatis* YZ03. This suggests that *R. wratislaviensis* YZ02 is more efficient in carbohydrate metabolism than *P. xanthosomatis* YZ03. These genetic insights not only enhance our understanding of these bacteria but also lay the groundwork for their potential biotechnological applications. The glycoside hydrolase family is a crucial enzyme in bacterial polysaccharide degradation and promotes bacterial growth and catalytic efficiency alongside CBM glycohydrolase-active enzymes (Hervé et al., 2010). Studies have shown that bacterial genomes are typically enriched in genes encoding glycoside hydrolases (Berlemont et al., 2015). Therefore, we analyzed the carbohydrate utilization ability of two bacterial strains using the Carbohydrate Active Enzymes Database (CAZy).^2^ The study found that *R. wratislaviensis* YZ02 and *P. xanthosomatis* YZ03 both possess genes encoding enzymes critical to the cellulose degradation pathway, including GH, GT, CE, PL, AA and CBM. *R. wratislaviensis* YZ02 and *P. xanthosomatis* YZ03 possess extensive GH family genes (Figure 7), which enable them to utilize a wide range of polysaccharides. This suggests that these strains have excellent cellulolytic capabilities. In addition, we identified genes encoding cellulases such as GH1 and GH5 as well as β-glucosidase genes (Koeck et al., 2014). Differences in gene number led to variations in the expression of CMCase, C1 and BGase between strains. These findings were consistent with our preliminary enzyme activity measurements, which indicated that *R. wratislaviensis* YZ02 had higher activity levels for all three enzymes compared to *P. xanthosomatis* YZ03. The synergistic action of these three enzymes plays an important role in the hydrolysis of cellulose and its derivatives to glucose (Henrissat et al., 1985). Additionally, we also identified genes encoding CBM family protein in both strains. These proteins can specifically bind polysaccharides, thereby increasing the catalytic efficiency of carbohydrase (Wei et al., 2022). Our results provide valuable genetic insights into the mechanisms of cellulose degradation by these bacteria, suggesting implications for their application in biomass conversion and waste recycling efforts. Our study is unique in identifying and characterizing these genetic attributes, thereby providing a comprehensive understanding of the cellulolytic mechanisms in *R. wratislaviensis* YZ02 and *P. xanthosomatis* YZ03.

## Data availability statement

The datasets presented in this study can be found in online repositories. The names of the repository/repositories and accession number(s) can be found in the article/Supplementary material.

## Author contributions

DM: Writing – original draft, Software, Writing – review & editing, Data curation, Formal analysis, Investigation, Methodology, Resources. HC: Data curation, Formal analysis, Investigation, Methodology, Software, Validation, Visualization, Writing – original draft, Writing – review & editing. DL: Data curation, Formal analysis, Investigation, Software, Validation, Visualization, Writing – original draft. CF: Data curation, Formal analysis, Investigation, Methodology, Project administration, Resources, Software, Validation, Writing – original draft. YH: Formal analysis, Investigation, Methodology, Software, Writing – original draft. TG: Data curation, Formal analysis, Investigation, Software, Writing – original draft. XG: Writing – original draft, Formal analysis, Investigation, Software. YZ: Writing – original draft, Data curation, Formal analysis, Investigation, Software. HW: Writing – original draft, Data curation, Methodology, Software. GT: Data curation, Formal analysis, Methodology, Software, Writing – original draft. HL: Writing – review & editing, Conceptualization, Data curation. KZ: Writing – review & editing, Conceptualization, Data curation, Formal analysis, Funding acquisition, Investigation, Methodology, Project administration, Resources, Software, Supervision, Validation, Visualization, Writing – original draft.

## Footnotes

^1^http://hmmer.org/

^2^http://www.cazy.org
