# Supplementary material for: Soil-derived cellulose-degrading bacteria: screening, identification, the optimization of fermentation conditions, and their whole genome sequencing
Source: Front Microbiol. 2024 Jul 10;15:1409697. doi: 10.3389/fmicb.2024.1409697 (PMC11266136; doi:10.3389/fmicb.2024.1409697)
Supplement: Supplementary file 1 [file Table_1.DOCX]

**Table S1 Name of the isolated bacterial strain**

| **Isolated codes** | **Isolated names** |
| --- | --- |
| BY02 | *Pseudomonas mosselii* BY02 |
| GY03 | *Pseudomonas putia* GY03 |
| BY03 | *Pseudomonas glycinae* BY03 |
| YZ02 | *Rhodococcus wratislaviensis* YZ02 |
| YZ03 | *Pseudomonas xanthosomatis* YZ03 |
| HJ01 | *Pseudomonas straminea* HJ01 |
| HJ02 | *Prestia qingshengii* HJ02 |
