# Supplementary material for: Soil-derived cellulose-degrading bacteria: screening, identification, the optimization of fermentation conditions, and their whole genome sequencing
Source: Front Microbiol. 2024 Jul 10;15:1409697. doi: 10.3389/fmicb.2024.1409697 (PMC11266136; doi:10.3389/fmicb.2024.1409697)
Supplement: Supplementary file 2 [file Table_2.DOCX]

**Table S2 Genome statistics for strains YZ02 and YZ03**

| Type | Number | |
| --- | --- | --- |
|  | **YZ02** | **YZ03** |
| GC content | 67.41 | 64.05 |
| Total gene length | 8,513,930 | 6,658,542 |
| Gene | 8,466 | 6014 |
| CDS | 8,355 | 5745 |
| 23S rRNA | 4 | 6 |
| 16S rRNA | 4 | 6 |
| 5S rRNA | 4 | 7 |
| Annotation | 8,315 | 5,703 |
| KEGG | 3,776 | 3,385 |
| Pathway | 2,129 | 1,878 |
| Nr | 8,308 | 5,703 |
| Uniprot | 8,307 | 5,683 |
| GO | 6,253 | 3,940 |
| COG | 7,080 | 4,758 |
| Pfam | 6,975 | 4,830 |
| Refseq | 8,256 | 5,694 |
| Tigerfam | 4,647 | 3,179 |
